# Licensed and Unlicensed NK Cells: Differential Roles in Cancer and Viral Control

**DOI:** 10.3389/fimmu.2016.00166

**Published:** 2016-05-02

**Authors:** Megan M. Tu, Ahmad Bakur Mahmoud, Andrew P. Makrigiannis

**Affiliations:** ^1^Department of Biochemistry, Microbiology and Immunology, University of Ottawa, Ottawa, ON, Canada; ^2^College of Applied Medical Sciences, Taibah University, Madinah Munawwarah, Saudi Arabia

**Keywords:** NK cells, licensing, Ly49, immunity, MHC-I

## Abstract

Natural killer (NK) cells are known for their well characterized ability to control viral infections and eliminate tumor cells. Through their repertoire of activating and inhibitory receptors, NK cells are able to survey different potential target cells for various surface markers, such as MHC-I – which signals to the NK cell that the target is healthy – as well as stress ligands or viral proteins, which alert the NK cell to the aberrant state of the target and initiate a response. According to the “licensing” hypothesis, interactions between self-specific MHC-I receptors – Ly49 in mice and KIR in humans – and self-MHC-I molecules during NK cell development is crucial for NK cell functionality. However, there also exists a large proportion of NK cells in mice and humans, which lack self-specific MHC-I receptors and are consequentially “unlicensed.” While the licensed NK cell subset plays a major role in the control of MHC-I-deficient tumors, this review will go on to highlight the important role of the unlicensed NK cell subset in the control of MHC-I-expressing tumors, as well as in viral control. Unlike the licensed NK cells, unlicensed NK cells seem to benefit from the lack of self-specific inhibitory receptors, which could otherwise be exploited by some aberrant cells for immunoevasion by upregulating the expression of ligands or mimic ligands for these receptors.

## Introduction

Natural killer (NK) cells are part of the innate immune system and were originally identified due to their unique ability to kill tumor cells without prior sensitization, which greatly differed from the defined functions of other major lymphocyte subsets (1, 2). The ability of an NK cell to recognize tumor or virus-infected cells is due to the expression of various activating and inhibitory receptors on its cell surface; such receptors include Ly49, CD94/NKG2, and NKp46, among others (3). While NK cell deficiencies are rare – possibly highlighting the necessity of NK cells in immunity – the few documented cases of NK cell deficiencies further support their important role. Individuals who exhibit reduced NK cell numbers, cytotoxicity, and/or cytokine production are characteristically more susceptible to certain viral infections (4–12).

Target cells can express ligands that bind to a variety of activating and inhibitory receptors on NK cells; it is this interplay between inhibitory and activating signals, which determines the NK cell response to the target (3). In addition, upon recognition of pathogen-associated molecular patterns, sentinels of the immune system, such as dendritic cells and macrophages, secrete a range of inflammatory cytokines in order to recruit and activate NK cells. Macrophages and dendritic cells are known producers of IL-2, IL-15, IL-18, and IL-21, all of which stimulate production of both type I and II IFNs by NK cells, as well as direct cytotoxicity of NK cells (13–17).

Members of the Ly49 receptor family, the murine functional homolog of the human killer-cell Ig-like receptor (KIR) family, can be either activating or inhibiting and interacting with class I major histocompatibility complex (MHC-I) molecules (18). Almost all adult nucleated cells constitutively express MHC-I molecules on their surface (19, 20). Both NK and CD8^+^ T cells depend on MHC-I recognition for their function. While the engagement of T cell receptors by MHC-I molecules is required for CD8^+^ T cell activation, the opposite is true for NK cells (21, 22). According to the “missing-self” hypothesis, NK cells preferentially target cells lacking MHC-I expression, which is recognized by self Ly49 receptors (23, 24). MHC-I expression on the target cell surface acts as a health marker for NK cells, signaling to the NK cell to spare the target. Conversely, aberrant cells often downregulate the surface expression of MHC-I to avoid detection and killing by cytotoxic T cells, but become a target for NK cells (25, 26).

The classical characterization of an NK cell is its ability to recognize and eliminate cells, which have decreased surface expression levels of MHC-I, a common phenomenon in cancer cells (2, 27, 28). Varied expression levels of human MHC-I –human leukocyte antigen (HLA) – on cells shows an inverse correlation with their susceptibility to killing by NK cells: variants with decreased levels of HLA were more susceptible to NK cell killing than the parental cell line, and accordingly, variants with higher levels of HLA benefited from protection from NK cells compared to the original cell line (29). While other inhibitory receptors, such as NKG2A, have been shown to play a role in NK cell licensing as well (30), the focus of our review will be on the dual role of the Ly49 family receptors on NK cells during target cell recognition and immune evasion.

## MHC-I-Mediated NK Cell Education/Licensing

Natural killer cell functionality depends on the presence of self-MHC-I molecules as proposed by the “licensing” hypothesis, in which a self-specific Ly49 receptor must interact with self-MHC-I in order for the NK cell to become functional (30, 31). Consequently, NK cells from *B2m*^−/−^ mice, in which MHC-I expression is abrogated, exhibit a diminished ability to kill MHC-I-deficient target cells that are normally readily killed by NK cells from WT mice, and exhibit defective cytokine production (28, 32). Similarly, our studies have demonstrated that the NK cells from Ly49-deficient mice are unlicensed and show impaired recognition of MHC-I-deficient target cells (33). NK cell responsiveness has also been shown to be proportional to the number of self-MHC-I-specific inhibitory receptors they express (34, 35). NK cells that express a higher number of self-MHC-I-specific Ly49 receptors are more responsive to stimuli than those that express fewer of these receptors. Human NK cells undergo a similar licensing process, which requires the interaction of HLA and KIR (36, 37). NK cells expressing self-MHC-I-specific KIRs exhibited a more robust responsiveness and cytokine production than self-KIR-negative NK cells.

While the mechanics of NK cell licensing are still unclear, whether it be *via* the arming, disarming, or rheostat model, or through *cis* interactions with self-MHC-I [as previously reviewed in Ref. (38)], the process has been shown to be an ongoing and fluid process, wherein an environmental change can alter the licensed state of even fully mature NK cells (34, 39–41). Contrary to the original school of thought that NK cell education occurs during NK cell development in the bone marrow, when unlicensed mature NK cells from MHC-I-deficient mice were adoptively transferred into WT mice, their function was restored showing that mature NK cell can also acquire licensing through the interaction of their inhibitory Ly49 receptors with the host MHC-I molecules (30, 31, 39, 40). Although licensing is important for NK cells to acquire effector functions, the unlicensed NK cells appear to respond effectively against target cells under specific conditions, such as when the target cells express high levels of MHC-I to evade NK detection by interacting with the inhibitory Ly49 receptors. This ability to detect MHC-I-expressing tumors and viruses may be the reason why up to 50% of NK cells are unlicensed with respect to self-Ly49 expression, but are still maintained in immune-competent mice (31, 42).

## Cancer Immunosurveillance by NK Cells

The importance of the immune system in tumor control is highlighted by the increased cancer risk in immune-compromised individuals. Those with human immunodeficiency virus (HIV) infection, including individuals who have progressed to acquired immunodeficiency syndrome (AIDS), are at notably greater risk of developing lung cancer independent of smoking (43). Immunosuppressed renal transplant patients have increased incidence of skin cancer over the general population (44). Those having undergone heart transplants are particularly at increased risk for non-Hodgkin’s lymphoma, oral, and lung cancers (45). Moreover, in human cross-sectional studies, the presence of tumor infiltrating lymphocytes is a strong predictor of positive patient outcome (46), indicating a correlation between the immune system and cancer protection or recovery.

In support of the importance of NK cells in cancer immunity, NK-compromised *beige* mice – a model for human Chediak–Higashi syndrome – exhibit defective cytotoxic activity against tumor cells, and are more susceptible to spontaneous fatal tumor development, possibly due to ineffective immunosurveillance (47, 48). Chediak–Higashi syndrome is caused by a homozygous or compound heterozygous mutation in the lysosomal trafficking regulator gene. Affected individuals present with a host of immunodeficiency disorders such as granular anomalies in their lymphocytes, defective chemotactic and bactericidal activity of their neutrophils, defective NK cell function, and defective peptide loading and antigen presentation (49–52). Antibody-mediated depletion of NK cells prior to tumor cell injection in various mouse strains results in prolonged tumor survival, as well as an increased number of artificial lung metastases and spontaneous metastases (53). In humans, NK cells comprise up to 15% of the blood lymphocytes (54). In a clinical setting, low NK cell activity in cancer-diagnosed individuals has been associated with poor prognosis, and those with advanced stage cancer often possess minimally cytotoxic NK cells (55). High cytotoxic activity of peripheral blood NK cells is correlated with up to 10% reduced incidence of cancer (56). As well, in a clinical case of childhood-onset Hodgkin’s lymphoma, this individual was observed to have non-functional NK cells (57).

## Recognition of MHC-I-Deficient Tumor Cells by Licensed NK Cells

Tumors have developed multiple mechanisms for evading host immune recognition. One well-documented escape mechanism, the downregulation of MHC-I expression, is effective against T cells, but renders the tumor more susceptible to NK cells. Reduced expression levels of MHC-I has been documented in bladder, breast, cervical, colorectal, and ovarian human cancers (58–63).

The classic tumor model, in which missing-self was first discovered retrospectively, also helps to highlight the importance of the MHC-I status of the target cell (23). Mutagenesis of RBL-5, a Rauscher virus-induced leukemia, led to the derivation of the MHC-I-deficient RMA-S and MHC-I-expressing RMA cell lines (23). The difference in MHC-I expression levels of these two cell lines leads to differential recognition by NK cells (23). The RMA-S induced flank tumors are much better controlled compared to the accelerated growth of the RMA tumors (23). With the use of *B2m*^−/−^ mutant mice, it has been shown that a MHC-I deficiency renders the NK cells defective at killing traditionally well recognized MHC-I-deficient NK tumor cell line targets (32).

**Figure 1:**
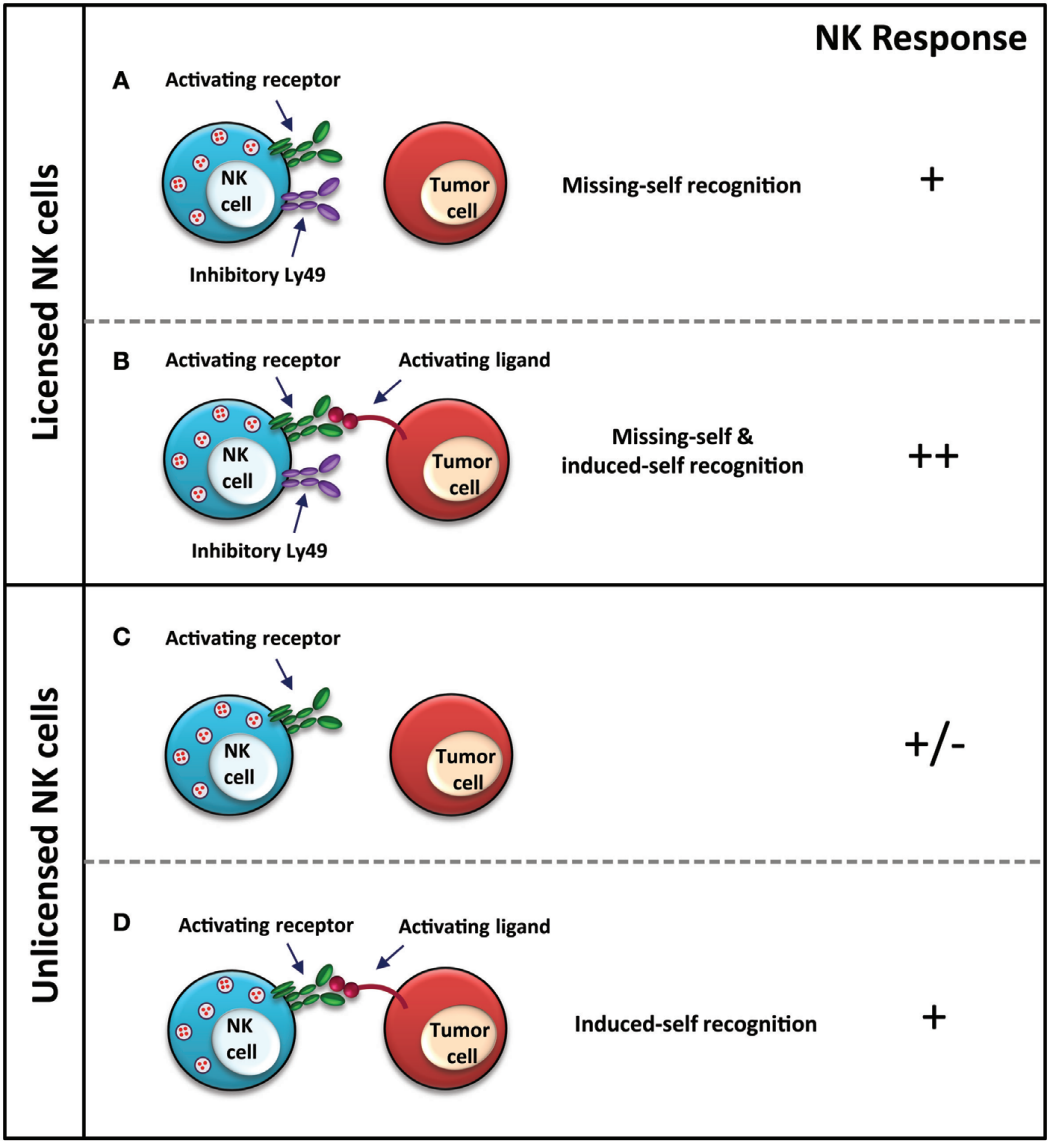
**NK cell response to MHC-I-deficient tumors**. Licensed NK cell recognition MHC-I-deficient tumors through “missing-self” due to a lack of MHC-I expression on the tumor cell **(A)**, as well as through “induced-self” *via* stress ligands, which are recognized by activating receptors on NK cells **(B)**. Unlicensed NK cells are unable to recognize MHC-I-deficient tumors through “missing-self” **(C)**, however, are still functional upon activating receptor–ligand interaction for “induced-self” recognition **(D)**. NK cell response “−” represents no activation, and “+” represents activation. In the case of **(C)**, +/− represents that the response of the NK cell, whether it is activated or not, also depends upon other immune cells, receptor–ligand interactions, and cytokines in the tumor microenvironment, which are not depicted in the figure for brevity.

Our group has shown that mice lacking Ly49-mediated NK licensing also exhibit reduced activity against MHC-I-deficient tumor cells both *in vitro* and *in vivo* (Figure 1) (33). In the *in vivo* rejection assays spanning up to 18 h, these mice exhibited reduced capacity at eliminating the MHC-I-deficient variants of RMA and C1498 compared to wild-type mice (33). Ly49-mediated NK cell education plays a major role in NK cell-mediated cancer immunosurveillance with tumor cell-induced flank tumors, experimental tumor metastases, methylcholanthrene-induced sarcoma, and spontaneous B cell lymphoma, with observations of increased and earlier onset tumor incidence in each model (64). Interestingly, the tumors, which developed in the mice with reduced levels of licensed NK cells, exhibited MHC-I-directed tumor immunoediting, wherein levels of both H-2K^b^ and H-2D^b^ were reduced, possibly as a mechanism of escape from cytotoxic T cells in an environment where evasion from NK cells is no longer a priority for survival due to their unlicensed nature (64).

## Induced-Self Recognition of Tumor Cells by Unlicensed NK Cells

While the self-specific inhibitory Ly49 receptors have been shown to be the mediators of NK cell licensing required for effective missing-self recognition (65), blockade of these receptors also helps to elicit a stronger NK cell response. Antibody-mediated blockade of self-specific Ly49 receptors improved *B2m*^−/−^ bone marrow allograft success similar to results seen with complete NK cell depletion; suggesting the importance of the inhibitory self-specific Ly49C/I subset in the effector functions of NK cells (66). This study shows successful grafting of MHC-I-deficient bone marrow cells in WT mice when Ly49C/I^+^ NK cells are depleted, thus providing evidence for missing self-recognition of MHC-I-deficient bone marrow cells by licensed NK cells.

Treatment of NK cells with monoclonal blocking antibody to Ly49C/I led to inhibited *in vitro* growth of MHC-I-expressing C1498, a murine leukemia cell line, and EL4, a T cell lymphoma cell line (65). *In vivo* antibody blockade prior to leukemia induction with C1498 led to increased survival (65). Considering everything we know about NK cell education and the importance of the Ly49C/I subset in licensing, in this case, the inhibitory nature of these receptors takes precedence over their educating role. However, one thing of note is the MHC-I status of the tumor cell lines utilized, with both lines expressing moderate to high levels of MHC-I, suggesting that missing-self recognition by licensed NK cell subsets is not a key requirement in this case. Self MHC-licensed NK cells are much less efficient than unlicensed NK cells at responding to the MHC class I-expressing target RMA cells (67). The requirement for licensed NK cells is not as imperative in the event that the tumor cells express MHC-I, since these cells would not traditionally be recognized through missing-self. Various cancer cells, which maintain expression of MHC-I on their cell surface, while escaping immune recognition through a missing-self response can also dampen NK cell activity through engagement of inhibitory KIRs (68–70). Antibody-mediated blockade of all KIR2D receptors elicits a heightened NK cell response with respect to cell cytotoxicity and has proven its efficacy in stage 1 clinical trials against acute myeloid leukemia and multiple myeloma (69–71).

**Figure 2:**
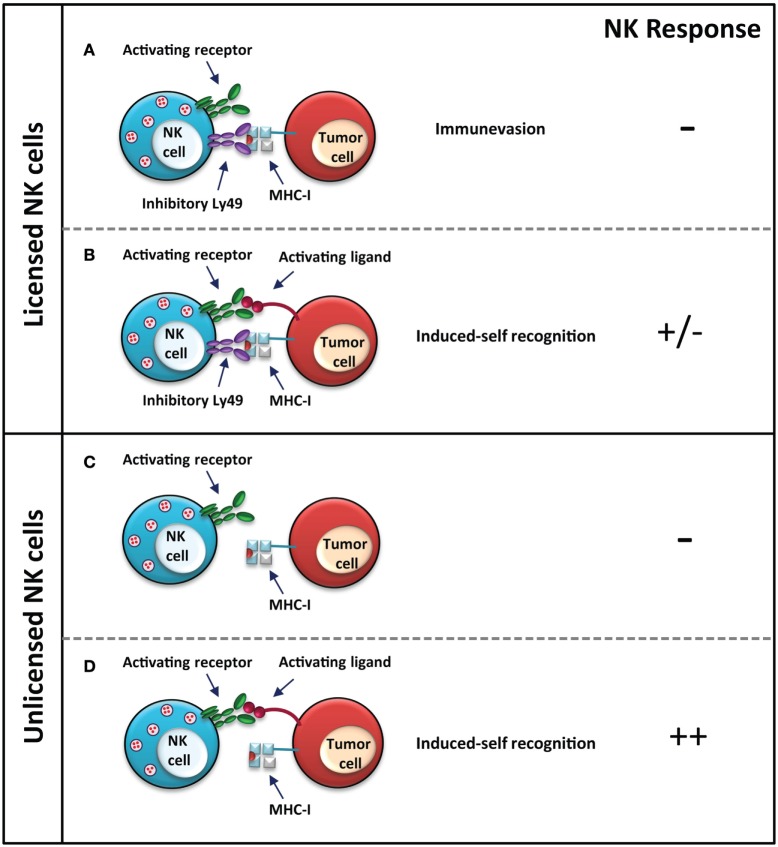
**NK control of MHC-I-sufficient tumors**. Licensed NK cells do not elicit a response against MHC-I-expressing tumor cells, since the presence of MHC-I is recognized as a health marker. Tumor cells have evolved various immune evasion mechanisms, such as in this case, to make them appear as a healthy “normal” cell to NK cells by presenting MHC-I; however, this may render them susceptible still to cytotoxic T cells **(A)**. The response to both activating and inhibitory signals, such as expression of both MHC-I and an activating stress ligand is dependent on a balance of activating and inhibitory signals, which will determine the response **(B)**. Unlicensed NK cells do not respond strongly to tumor cells, which express MHC-I **(C)**; however, a stronger response is elicited if the tumor cell expresses stress ligands recognized by the activating receptor **(D)**. In the case of unlicensed NK cells, the response is stronger since the activating signal is not dampened by inhibitory signals *via* inhibitory Ly49–MHC-I interaction as in the licensed NK cells. NK cell response “–” represents no activation, and “+” represents activation.

In some instances, it appears that the unlicensed NK cells are more efficient at eliminating MHC-I-expressing aberrant cells (Figure 2). Unlicensed human NK cells, which lack inhibitory self-KIR expression, are more effective at killing neuroblastoma cells through antibody-dependent cell-mediated cytotoxicity (ADCC), following treatment with an antibody, which targets the disialoganglioside surface antigen GD2 on tumor cells (72). The activated NK cells recognize MHC-I on the cell surface as a health marker, thus sparing the MHC-I-expressing neuroblastoma cells and concurrently selecting for the MHC-I-expressing subset. MHC-I-expressing tumor cells can inhibit licensed NK cells through the engagement of inhibitory KIRs. Unlicensed NK cells, on the other hand, are not inhibited and are better mediators of neuroblastoma cell killing *via* ADCC, which is particularly relevant in the absence of tumor-expressed NK activating ligands (72). Therefore, unlicensed NK cells appear to be the better mediators of an anti-tumor response when the tumor cells express ligands for self-specific inhibitory NK cell receptors.

Natural killer cells can also kill certain virus-infected and tumor cells despite their expression of MHC-I, as explained by the “induced-self” model (73–75). The licensed status of the NK cell, in this case, does not wholly dictate its response. Several studies have shown that unlicensed NK cells can recognize aberrant cells through the recognition of activating ligands, similar to licensed NK cells (Figures 1 and 2). The NKG2D activating receptor plays a major role in the control of both lymphoid and non-lymphoid cancers; loss of this receptor leads to increased susceptibility of oncogene-driven cancer development (76). Expression of Rae1, the ligand for the activating NKG2D receptor, on RMA cells elicits a strong *in vivo* rejection response by unlicensed NK cells in Ly49-deficient mice (33). *In vitro* killing of splenocytes from Rae1ε transgenic mice is comparable between licensed and unlicensed NK cells, indicating no effect of licensing in this model (33). Additionally, *in vitro* and *in vivo* killing of Rae1β-expressing RMA-S cells is comparable between NK cells from wild-type mice and unlicensed NK cells from *B2m*^−/−^ and Ly49-deficient mice, suggesting that other signals, such as those from activating receptors, are able to compensate for the hyporesponsiveness of unlicensed NK cells to the loss of MHC-I expression (33, 64). As well, stimulation with the double-stranded RNA viral mimic, polyinosinic:polycytidylic acid (poly I:C), induces a strong immune response in the otherwise hyporesponsive Ly49-deficient mice against MHC-I-deficient B16 F10 tumor cells. Prior treatment with poly I:C improves tumor rejection, reducing the number of pulmonary metastases in Ly49-deficient mice to wild-type levels (64). While the unlicensed NK cells are hyporesponsive in an Ly49-dependent manner, NK activation can be achieved through other means, which can bypass the hyporesponsiveness of these cells.

## NK Cell-Mediated Recognition of Virus-Infected Cells

The importance of NK cells in an immune response against a pathogen challenge can be seen in various clinical case studies of individuals lacking functional NK cells, leading to recurrent, life-threatening infections by otherwise non-consequential pathogens (4, 77). In humans, NK cells have been shown to play a seminal role in the control of viruses from the herpesvirus, poxvirus, and papillomavirus families (4, 77). Murine studies have further established the importance of NK cells in protection against vaccinia, hepatitis, and cytomegalovirus infections (78). In one study, anti-asialo GM1 antibody-mediated depletion of NK cells followed by viral infection lead to increased viral titer and interferon (IFN) production, as well as increased viral-associated hepatitis and liver damage (78).

Viruses have evolved mechanisms to evade recognition by immune cells. The MHC-I antigen presentation machinery appears to be a major target for immunoevasion by viruses. Murine cytomegalovirus (MCMV) gene products, such as m06, m152, and m04, have the ability to alter MHC-I function (79–82). While *m06* and *m152* gene products interfere with the expression of assembled MHC-I molecules on the cell surface, *m04* gene product does not interfere with cell surface expression, but rather prevents MHC-I recognition by T cell receptors (79–82). These strategies as well as the expression of an MHC-I mimic (described below) prevent recognition of infected cells by T cells and at the same time maintain sufficient expression of ligands for inhibitory Ly49 receptors to prevent “missing-self” recognition by NK cells. Similarly, an increased binding of inhibitory KIR by MHC-I ligands on infected cells is reported during influenza virus infection (83, 84). In these studies, a redistribution of MHC-I molecules on the infected cells was proposed to allow better recognition by the inhibitory KIR and subsequent NK cell inhibition (84). Infection of a human pulmonary epithelial cell line by influenza virus was also shown to activate p53 and cause MHC-I upregulation on the surface of infected cells (85).

**Figure 3:**
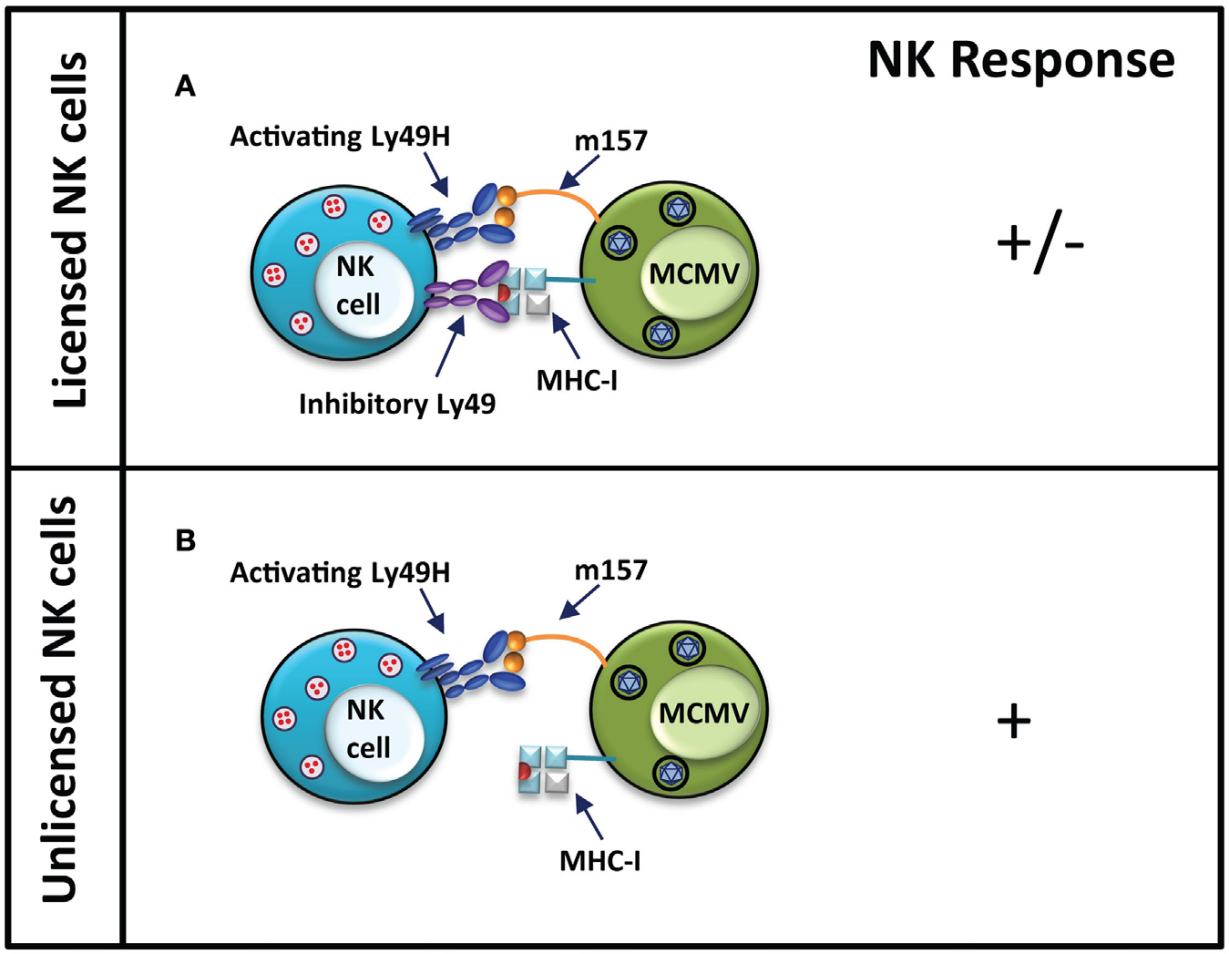
**Unlicensed NK cells dominate responses to MCMV infection in MCMV-resistant mouse strains**. In MCMV-resistant mouse strain, NK cells activation occurs when the activating Ly49H receptor is engaged by m157 protein on the infected cells. Simultaneous engagement of inhibitory Ly49 receptors by MHC-I molecules can inhibit activation of licensed NK cells **(A)**. In unlicensed NK cells, the interaction of Ly49H and m157 induces a strong activating response due to the lack of self-MHC-I-specific inhibitory Ly49 receptors on these cells **(B)**. NK cell response “−” represents no activation, and “+” represents activation.

Viruses have been shown to also utilize other mechanisms, such as mimicry of host proteins on their cell surface, to evade host immune recognition. Often these viral mimics engage inhibitory receptors on immune cells to cause inhibition of immune responses. A classical example of this is the product of MCMV *m157* gene, a viral MHC-I-like protein expressed on the surface of infected cells (86, 87). While similar to MHC-I in structure, expression of the viral mimic is independent of the host’s MHC-I antigen processing machinery (88). Engagement of inhibitory Ly49 receptors by m157 molecules on the surface of infected cells causes NK cell inhibition in MCMV-susceptible mouse strains (Figure 3) (89). Leukocyte Ig-like receptor 1, also known as ILT2, is a cell surface receptor expressed on human immune cell subsets including T and NK cells, and is capable of binding classical MHC-I and HLA-G (90). A mechanism of immune evasion by human cytomegalovirus involves the expression a glycoprotein human homolog of MHC-I, UL18, which can inhibit NK cell function by binding to its ILT2 receptor (91, 92).

Nevertheless, NK cells are able to recognize virus-infected cells through the engagement of their activating receptors. Activating receptors on NK cells are thought to have arisen as a result of selective pressure exerted by continuous pathogen challenge. Following Epstein–Barr infection, increased expression of a MHC-I ligand, which interacts with the activating KIR2DS1, is detected on the cells surface (93). In MCMV-resistant mouse strains, such as C57BL/6 (B6), the activating Ly49H receptor is able to recognize the MCMV m157 glycoprotein (86, 87). The presence of the activating Ly49H receptor in the Ly49 receptor repertoire of B6 mice confers resistance from MCMV infection. In contrast, strains, which lack genes encoding for the activating Ly49H, such as 129 and BALB/c, or the genetically manipulated Ly49H-deficient B6, are highly susceptible to MCMV infection (86, 87, 94). In the 129 mouse strain, m157 binds the inhibitory Ly49I receptor (86). Sequence variants of m157 are known to also interact with the inhibitory Ly49C receptor from B6 mice, which could inhibit NK cell activation. However, since most Ly49H^+^ NK cells do not express Ly49C, MCMV clearance is not disrupted in B6 mice (95, 96). Similarly, the activating Ly49P receptor confers resistance in MA/My mice to MCMV infection through the recognition of MCMV m04 protein in association with H-2D^k^ MHC-I haplotype (97, 98). In both cases, viral gene products aimed at evading immune recognition have become the target for activating Ly49 receptors.

## Is NK Cell Licensing Always Required for the Recognition of Virus-Infected Cells?

The fact that the same inhibitory receptors, which are exploited by viruses to evade immune recognition, are also involved in NK cell licensing raises the question of whether NK cell licensing is always required for the recognition of virus-infected cells. As it appears, licensed NK cells, bearing inhibitory Ly49 receptors, are at a disadvantage during certain virus infections. It has been shown during MCMV infection that the NK-mediated response is dominated by the unlicensed (Ly49C/I^−^) NK cell subset (67). While the antibody used to deplete the licensed NK cells, 5E6, is expected to deplete both Ly49C and Ly49I, there is also evidence that 5E6 is only capable of recognizing Ly49I on NK cells (99, 100).

In contrast to licensed NK cells, which are the primary mediators of an anti-tumor response through missing-self recognition, it is the unlicensed cell subset, which confers protection from MCMV infection. Selective depletion of the Ly49C/I^+^ licensed NK cell subset rendered minimal viral titer increase, while depletion of Ly49C/I^−^ unlicenced NK cells led to significant viral titer increase in the infected mice. Moreover, adoptive transfer of the Ly49H^+^ Ly49C/I^−^ NK cell subset into neonatal mice was sufficient to protect them against an MCMV infection, and was more effective than the Ly49H^+^ Ly49C/I^+^ NK cell subset. The interaction between Ly49C/I and its ligand limits the ability of licensed NK cells to control the infection. These findings indicated that unlicensed NK cells are responsive and play a major role in an immune response during a viral infection, the activity of unlicensed NK cells could be due to the pro-inflammatory cytokine environment of viral infection, which is known to enhance NK cell function. As a further confirmation, the classical unlicensed NK cell model – *B2m*^−/−^ mice – which possess only unlicensed NK cells due to their lack of MHC-I surface expression, was better able to control MCMV infection than their wild-type counterparts (67). NK cell depletion in *B2m*^−/−^ during MCMV infection leads to enhanced virus titer in the salivary gland (80). Our group has also shown that Ly49-deficient and *B2m*^−/−^ mice, which possess unlicensed NK cells, fare better following influenza infection than their wild-type counterparts in an NK cell-dependent manner (101). *B2m*^−/−^ and Ly49-deficient mice treated with NK cell-depleting monoclonal antibody, as well as perforin-deficient Ly49-deficient mice, are more susceptible to influenza virus infection, demonstrating that the improved survival of these mice is due do the functional activity of unlicensed NK cells. The Ly49-deficient mice exhibit reduced viral titer and lung pathology. Our study also provides evidence for influenza virus infection-driven immune evasion. Following influenza virus infection, upregulation of MHC-I is observed on pulmonary epithelial cells, possibly as a mechanism to evade detection by licensed NK cells. This provides Ly49-deficient mice with an advantage since their unlicensed NK cells will not be affected by the increased levels of MHC-I, which would traditionally be recognized by members of the Ly49 receptor family leading to NK cell inhibition. Moreover, blockade of the interaction between Ly49:MHC-I rendered licensed NK cells in wild-type mice better at controlling influenza virus infection. It is suggested that the unlicensed NK cells are better effectors in viral control due to their ability to surpass inhibition mediated by MHC-I or MHC-I-like viral ligands expressed on the surface of infected cells.

In a hematopoietic stem cell transplantation (HSCT) mouse model for NK cell licensing, depletion of licensed NK cells resulted in higher viral titers in the liver of MCMV-infected mice at early time points but not at later time points after infection (102). In the same study, the licensed NK cells also expanded and produced IFNγ upon infection but were suppressed by regulatory T (Treg) cells and TGF-β (102). It was proposed that the licensed NK cells mount a strong early response against MCMV infection, but become inhibited or exhausted. The unlicensed NK cells, on the other hand, show a strong late response due to the presence of activation stimuli and the absence of inhibition from the inhibitory Ly49:MHC-I binding. This is also corroborated by results showing that prior activation by stimuli, such as poly I:C, results in an efficient anti-virus and anti-tumor response by the unlicensed NK cells (64, 66, 102).

Epidemiological human studies have shown that HIV-infected individuals who have KIR3DS1, an activating NK cell receptor, and its ligand, HLA-B Bw4-80I, exhibit slow progression to AIDS, compared to other HIV-infected individuals (103). In support of this finding, an *in vitro* study has shown that NK cells derived from individuals with the KIR3DS1/HLA-B Bw4-80I compound genotype were able to mediate inhibition of HIV-1 replication in a contact-dependent manner (104). Interestingly, early after HIV infection, the frequencies of the activating KIR3DS1^+^ and the inhibitory KIR3DL1^+^ NK cells are specifically increased in patients with acute HIV-1 infection in the presence of HLA-B Bw480I. Unfortunately, this expansion is not associated with reduction in HIV levels in the blood. Engagement of the inhibitory KIR3DL1 receptor on these NK cells with its ligand on the target cells could result in the inhibition of NK cell cytotoxicity toward the HIV-infected cells, explaining the maintained level of HIV in those patients in comparison to KIR3DL1-deficient patients (105). Similarly, studies have shown that CD56^−^ CD16^+^ NK cells, which are greatly expanded in HIV-viremic individuals, have impaired function. Characterization of this NK cell subset revealed that the expression of inhibitory KIR2DL2 and KIR2DL3 receptors were high on these cells, which would explain their defective lytic capability toward HIV-infected cells (106). Taken together, these reports indicate that HIV-infected cells may augment NK cell inhibition through interactions between inhibitory KIR and HLA receptors.

Therefore, under circumstances where inhibitory Ly49 receptors are engaged strongly by MHC-I or viral mimic ligands on the infected cells, NK cells that lack inhibitory receptors for MHC-I seem to exhibit better effector functions. This may also explain why a large proportion of unlicenced NK cells, which do not express receptors for self-MHC-I, are maintained in both mice and humans. In mice, up to half of the NK cells are “unlicensed” with respect to self-Ly49 expression (31, 42). In human studies, almost 25% of CD56^dim^ and over 60% of CD56^bright^ NK cells do not express KIR, as assessed by their negative staining for KIR2DL1, KIR2DS1, KIR2DL2, KIR2DL3, KIR2DS2, KIR3DL1, and KIR2DS4 (107).

## Conclusion

These non-conventional observations possibly underlie a different approach to understanding NK cell function dependent on its licensed or unlicensed status. The licensed status of the cell is of biological importance during rejection of MHC-I-deficient cancer cells, MHC-mismatched bone marrow transplants, and other target cells which exhibit MHC-I downregulation. Various studies suggest that licensed NK cells excel at tumor cell recognition and, while still controversial, the unlicensed NK cells preferentially protect from viral infections. The roles of these NK cell subsets may not be so clearly defined as either preferential to viral or cancer control, but may be due to more nuances of the disease. Viral infection induces robust cytokine secretion, which may reactivate unlicensed NK cells to respond to the infection. Additionally, the status of NK cell control may not be dependent on the cells being licensed or unlicensed, as it has been shown that activation can happen in the absence of licensing through other means such as through engagement of activating receptors such as NKG2D, ADCC, or through immunostimulants such as poly I:C. Rather, NK recognition may be dependent on the characteristics of the aberrant cell, including its MHC-I expression levels and the microenvironment where they are encountered. The role for unlicensed NK cells in the control of cancers and virally infected cells, which have mediated upregulation of MHC-I helps to explain why the unlicensed NK cell subset is still present in modern day mice and humans, and has not been evolutionarily selected against.

## Author Contributions

MMT and ABM wrote the manuscript. APM supervised and reviewed the manuscript.

## Conflict of Interest Statement

The authors declare that the research was conducted in the absence of any commercial or financial relationships that could be construed as a potential conflict of interest.
